# Reducing Blast Furnace Dependence on Coke: A Review of Low-Carbon Strategies

**DOI:** 10.1007/s11837-026-08659-x

**Published:** 2026-08-17

**Authors:** Zhanjun Wang, Tongtong Zhang, Yulong Ding

**Affiliations:** Birmingham Centre for Energy Storage and School of Chemical Engineering, University of Birmingham, Birmingham, B15 2TT UK

## Abstract

Reducing coke consumption is one of the most effective approaches for lowering CO_2_ emissions in blast furnace (BF) ironmaking. Coke plays both chemical and physical roles in the BF, acting as a reductant, providing heat, and supporting the burden to maintain gas permeability. While part of its chemical function can be replaced by alternative carbon-based fuels and reducing agents, its physical load-bearing role remains difficult to substitute. This review summarizes recent studies on the partial replacement of coke in BFs from the perspective of low-carbon ironmaking. Rather than treating different coke-saving technologies as separate methods, this review discusses coke reduction according to the specific functions of coke being replaced. Alternative carbon sources, H_2_-rich reductants, CO_2_-to-CO recycling, load-bearing packed-bed materials, and burden structure optimization are considered. This perspective helps explain why alternative fuels, H_2_-rich gases, and recycled CO can reduce the chemical demand for coke, while deeper coke reduction remains limited by the difficulty of replacing coke’s structural role. Overall, meaningful coke reduction is more likely to rely on the combined use of several approaches while maintaining process stability and engineering feasibility. This function-based understanding may help guide the development of more practical lower-carbon BF ironmaking strategies.

## Introduction

Over the past three decades, global energy-related CO_2_ emissions have risen sharply, increasing from 22.6 billion tonnes in 1996 to more than 38 billion tonnes in 2025, largely driven by energy combustion and industrial processes.^1^ The steel industry contributes approximately 8–9% of global CO_2_ emissions, with the blast furnace–basic oxygen furnace (BF-BOF) route remaining the dominant production pathway.^2–4^ The BF-BOF process generates around 1.8 t CO_2_ per ton of steel, with over 80% of the total CO_2_ emissions arising from the BF ironmaking stage.^5–8^ The reduction of BF carbon emission is therefore urgent for combating global warming challenges.^9,10^

Coke plays an essential role in conventional BF operation, serving three primary functions. First, it serves as the primary reductant and heat source (chemical function). Coke reacts with iron oxides to form Fe (either or both direct and indirect) and the combustion of coke through the tuyere region creates a high-temperature environment, providing heat for the reduction reaction in the BF.^11^ Meanwhile, carbon and CO_2_ also react to produce CO, which reduces iron oxides.^12,13^ In recent decades, technologies have been developed/under development to replace part of coke’s chemical function by using auxiliary fuels, including pulverized coal, natural gas, H_2_-rich gases, and biomass fuels.^14–18^ More recently, CO_2_ capture, conversion and utilization strategies, such as CO_2_ electrolysis and CO_2_ splitting using perovskite-based oxygen carriers,^19–23^ have been proposed to regenerate CO from the captured CO_2_ and re-inject it into the BF. Under the premise that CO_2_ can be efficiently converted into CO and recycled to participate in indirect reduction reactions in the BF, the chemical function demand for coke has a great potential to be substantially reduced. Second, coke serves as a carburizer. The dissolution of carbon into the hot iron metal is essential for stable production, typically resulting in pig iron containing 4–4.5 wt.% C.^24,25^ Third, coke serves critical physical and structural support. It maintains gas permeability through its porous bed and acts as a stabilizer in the cohesive zone where softening and melting of iron-bearing materials occur.^26,27^ Even as gaseous reducing agents (CO and H_2_) increasingly replace coke’s chemical duties, the BF still requires a robust, high-temperature-resistant internal structure to ensure mechanical stability and permeability.^28,29^ These physical functions remain the most difficult to replace. Thus, the way forward is not simply to address the challenges of the coke elimination but also to explore potential materials and operational strategies to partially replace the load-bearing function of coke. Such materials should not melt, incur excessive undesired reactions, or generate unwanted by-products under BF conditions. This issue represents an important long-term research challenge for deeper coke reduction in BF ironmaking.

This paper aims to systematically review recent efforts toward the partial replacement of coke in BFs for low-carbon ironmaking. The purpose of this review is not to present the chemical and structural functions of coke as a new concept, since these roles are already well recognized in BF ironmaking. Rather, these functions are used as an analytical framework for critically assessing the real substitution potential of different coke-reduction strategies. Many existing studies discuss auxiliary fuel injection, hydrogen enrichment, biomass use, carbon recycling, and burden optimization as separate approaches. However, these routes target different functions of coke and therefore have different limitations. For example, alternative carbon sources and H_2_-rich gases can reduce the chemical demand for coke, but they do not directly replace the load-bearing and permeability-maintaining function of coke in the cohesive zone and lower furnace. Conversely, potential load-bearing materials may address the structural role of coke, but their feasibility is still highly uncertain because of slag attack, abrasion, cost, recyclability, and compatibility with dynamic BF operation. Therefore, this review evaluates coke-reduction strategies by considering not only their carbon-saving potential but also the coke function targeted, industrial constraints, and possible influence on furnace stability. This distinction does not imply that the chemical and structural roles of coke can be physically separated in real BF operation. In the cohesive zone and lower furnace, these roles are strongly coupled: solution-loss reactions consume carbon and weaken coke strength and permeability, while coke layers control gas flow, liquid drainage, heat transfer, and carburization. This framework helps explain why moderate coke reduction can be achieved through several mature technologies, whereas deeper coke reduction remains limited by the difficulty of replacing the structural role of coke. Based on this assessment, the review identifies practical near-term routes and longer-term research needs for lower-carbon BF ironmaking.

This article is structured in the following manner. The "Introduction" section above introduces the background to low-carbon BF ironmaking and the challenge of reducing coke dependence while maintaining furnace stability. The "Technical Routes for Reducing Coke Dependence of BFs" section summarizes the main strategies for partial replacement of coke functions, focusing on alternative carbon sources, H_2_-rich reduction, and potential load-bearing materials. The "Burden, Furnace Structure, and Operational Optimization as Indirect Coke-Saving Routes" section discusses burden, furnace structure, and operational optimization as indirect coke-saving routes. The "CO_2_-to-CO Conversion as an Enabling Route for Low-Coke BF Operation" section introduces CO_2_-to-CO conversion technologies as an enabling approach for carbon recycling within BF system. The "Overall Comparison of Coke-Reduction Strategies" section provides an overall comparison of coke-reduction strategies. Finally, key conclusions and future perspectives are presented in the "Conclusions and Perspective" section.

## Technical Routes for Reducing Coke Dependence of BFs

**Fig. 1:**
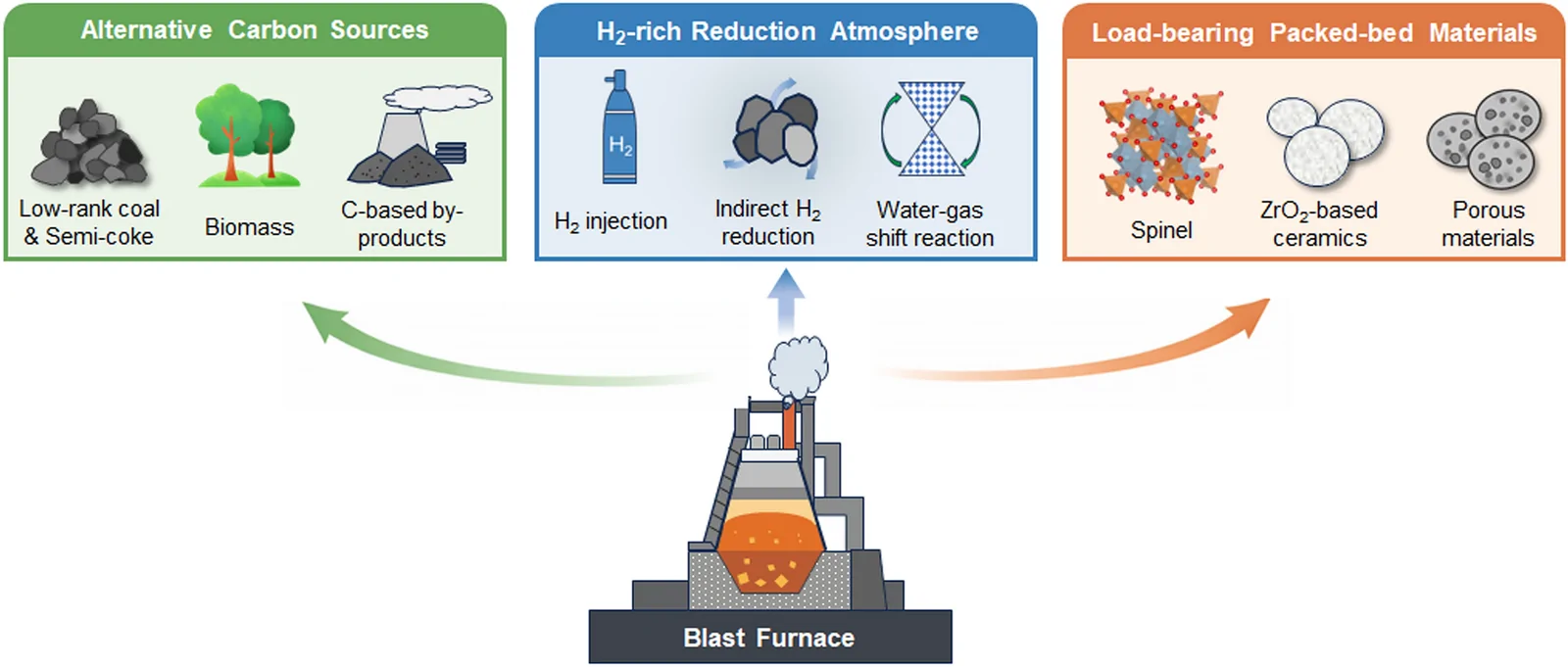
Main approaches for reducing coke consumption and structural dependence in BF ironmaking.

Since coke performs both chemical and physical roles, most studies focus on partially replacing specific functions rather than eliminating coke entirely. Existing approaches can be generally divided into three categories (see Fig. 1): (1) alternative carbon sources to provide part of the carbon input, (2) H_2_-rich reduction atmospheres, where H_2_-bearing gases directly decrease the demand for carbon-based reduction, and (3) load-bearing packed-bed materials that may help maintain burden structure and permeability. Among these routes, the first two mainly target the chemical demand for coke, whereas the third attempts to address its much harder-to-replace structural role.

### Alternative Carbon Sources

#### Low-Rank Coal and Semi-coke

Low-rank coals and semi-coke powders usually exhibit high oxygen content and well-developed porosity, which lead to significantly higher CO_2_ reactivity after carbonization compared with conventional BF coke.^30–34^ Considerable efforts have been made to upgrade these materials prior to briquetting. For example, Mollah et al.^35^ showed that hydrothermal dewatering combined with acid washing reduced oxygen functionality and enhanced structural ordering of brown coal. After hot briquetting (150–230°C) and carbonization at 1000°C, the resulting products showed strength close to that of BF coke, and exhibited lower surface area and reactivity compared to untreated coal-derived chars, which was attributed to the development of a more graphitic structure. In a later study, the same group found that alkali treatment followed by hot briquetting produced low-surface-area carbon materials with improved mechanical integrity.^36^ However, even after these treatments, the CO_2_ reactivity remained higher and the graphitic ordering remained lower than that of standard BF coke. This suggests that such briquette cokes may be suitable for partial replacement, but are still difficult to use as a full substitute for coke in BFs.^37^

In addition to these pretreatment methods, semi-coke fines from low-rank coal pyrolysis have also been consolidated using carbonated consolidation processes. Guo et al.^38^ reported that optimization of the briquetting pressure to 93.63 MPa, together with Ca(OH)_2_-assisted carbonation, produced coke with good compressive strength, acceptable heating value, and reduced SO_2_/NO_*x*_ emission. Therefore, it is clear that pretreatment methods directly affect pore development, surface area, and CO_2_ reactivity, which are critical factors for stable BF operation. However, even under such modification, low-rank coal-based-formed coke still cannot match the hot strength of traditional coke around 1200–1400°C. The main reason lies in its insufficient plastic phase formation and less optimized meso-/macropore structure during carbonization.^35,36^ Overall, these studies show that low-rank coal and semi-coke are more suitable as partial carbon substitutes than as direct replacements for the high-temperature structural function of coke. Their main value lies in reducing fossil carbon input, but their weaker hot strength remains a key limitation for further substitution.

#### Biomass-Based Materials

Biomass-based carbon materials, including wood chips, straw, and other agricultural and forestry wastes, have been explored as renewable substitutes for fossil-based coke in BF operation to reduce net CO_2_ emissions.^39,40^ The study by Seo et al.^41^ showed that biomass–coal mixtures could produce bio-coke with calorific values exceeding 7000 kcal/kg and ignition temperatures in the range of 400–600°C, while maintaining the basic fuel requirements. However, Florentino-Madiedo et al.^42^ reported that biomass addition often lowers coal blend fluidity and weakens thermoplastic behavior, unless suitable binder systems or pre-treatment methods are applied. Thus, pretreatment plays a critical role in improving structural performance. For example, Wibawa et al.^43^ found that binderless hot briquetting of torrefied woody biomass followed by carbonization at 1000°C produced formed coke with indirect tensile strengths ranging from 8 MPa to 32 MPa, depending strongly on torrefaction temperature and particle size. An optimal torrefaction temperature at around 275°C was found to enhance interparticle bonding and densification during carbonization, suggesting that biomass pretreatment is essential for achieving acceptable mechanical properties.^43^ More recently, biomass upgrading routes combining hydrothermal carbonization and pyrolysis have also been proposed to produce higher-quality BF injection fuels, with reduced alkali metal content, ash content, and specific surface area, as well as improved carbon ordering and gasification behavior.^44^

At the industrial scale, biomass-containing briquettes have demonstrated technical feasibility. Mousa et al.^45^ reported in BF trials that charging 55% bio-briquettes (~ 64 kg/tHM) reduced carbon consumption by ~ 10 kg/tHM and lowered total CO_2_ emissions by 33–40 kg/tHM, without significant deterioration in shaft permeability or gas utilization. The process model conducted by Orre et al.^46^ further indicated that combining the biomass injection with the top charging strategy can reduce CO_2_ emissions by up to 30–35%, while maintaining stable operation of the furnace. Recent feed-flexible BF concepts further suggest that tailored biochar may be used in different forms, including tuyere injectant, biocoke blend component, and limited top-charged lump biochar, although each route remains constrained by material quality, availability, and furnace stability.^47^

Nevertheless, biomass-based carbon materials generally exhibit higher intrinsic reactivity and a lower degree of graphitization than conventional BF coke. As a result, their resistance to high-temperature degradation in the cohesive zone remains limited, restricting their role primarily to partial substitution rather than full structural replacement.^48^ Therefore, the main significance of biomass-based materials is not that they can replicate coke performance, but that they can lower the net carbon footprint of BF ironmaking when introduced in controlled proportions.

#### Carbon-Bearing Wastes

Carbon-bearing waste materials from both steel plants and other waste sources provide an additional route for reducing fossil coke consumption. Residues generated within steel plants, including coke breeze, BF dust and sludge, etc., contain various amounts of carbon.^3,49–52^ Cold-bonded briquetting is widely adopted to convert these materials into mechanically stable agglomerates.^34^

Li et al.^53^ studied reduction kinetics of cold‐bonded briquettes prepared from return fines of sinter with carbon monoxide and coke, and observed that briquettes containing coke often exhibit a higher reduction velocity index and a lower apparent activation energy (32.4 kJ mol^−1^) compared with coke-free briquettes (38–57 kJ mol^−1^), leading to more complete phase transformation and improved metallurgical performance. For briquettes prepared from BF dust or sludge, self-reduction can start at around 500°C and is nearly complete by 1200°C. During heating, the decomposition of hydrates and carbonates generates gaseous intermediates that promote carbon gasification and accelerate the stepwise transformation of iron oxides.^54^ In addition, Fe-bearing process wastes can also be recycled through RHF-based composite pellets to produce DRI for BF use. Chung et al.^52^ reported that pellets prepared from FINEX process wastes and anthracite achieved over 84% oxygen removal at 1300°C, indicating the potential of converting integrated steel plant wastes into recyclable BF feedstock, although problems such as incomplete reduction at lower temperatures, pellet bursting, and impurity-related operational problems still need to be addressed. Coke breeze has also been improved through pitch-bonded briquetting followed by carbonization, producing formed coke with compressive strengths above 30 MPa and enabling partial substitution of metallurgical coke in BF operation.^55,56^ Diez et al.^57^ reported that briquetted coal-tar sludge and oily residues could be added to coking blends at up to 10 wt.% in coke ovens, and when binder type and particle size were optimized, coke reactivity and post-gasification strength remained largely unchanged, allowing coke quality to meet BF requirements. Waste plastics can also serve as supplementary carbon sources, mainly through tuyere injection. Their high hydrogen content supports combustion and indirect reduction, but the low mechanical strength, low softening temperature, and poor gas permeability limit their application in large proportions.^58,59^ Recent flash-pyrolysis and burnout studies of plastic- and paper-derived refuse fuels further indicate that such alternative reductants must be evaluated under raceway-relevant heating rates and short residence times before large-scale BF injection.^60^

These studies show that carbon-bearing wastes can provide carbon and help reduce landfill disposal. However, recycling carbon-bearing wastes requires the careful control of impurity accumulation, thermal decomposition behavior, and possible effects on slag chemistry, so as to avoid negative effects on furnace permeability and slag quality.^61^ Compared with low-rank coal and biomass, the key advantage of carbon-bearing wastes is circular resource utilization. However, their larger compositional variation and impurity burden make process control more critical. In practice, their role is again mainly chemical substitution rather than replacement of the structural function of coke.

For practical BF operation, the use of carbon-bearing wastes should also be assessed from the viewpoint of long-term impurity accumulation. Elements such as alkali metals, Zn, Pb, S, P, and Cl, as well as ash-forming oxides, may circulate or accumulate in the furnace and influence burden degradation, gas permeability, slag chemistry, viscosity, melting behavior, and slag–metal separation.^51,62^ Therefore, the suitability of such wastes should be evaluated not only by their carbon content and calorific value, but also by impurity mass balance, high-temperature softening/melting behavior, slag–property changes, and long-term pilot- or plant-scale monitoring.

### H_2_-Rich Reduction Atmospheres

Partial replacement by H_2_ in the reduction process can weaken the solution loss reaction and reduce coke degradation in the lower part of the BF. In this way, the structural demand for coke can also be lowered to some extent by changing the furnace atmosphere.^63–65^ This route does not replace coke physically, but reduces the rate at which coke is consumed and degraded, thereby indirectly preserving its supporting role.

#### Reducing Coke Solution Loss in the BF

Coke degradation in the BF is largely governed by the following carbon solution loss reactions:^66–68^

1 $$ {\mathrm{C}}\left( {\mathrm{s}} \right) + {\mathrm{CO}}_{{2}} \left( {\mathrm{g}} \right) = {\mathrm{2CO}}\left( {\mathrm{g}} \right) $$

2 $$ {\mathrm{C}}\left( {\mathrm{s}} \right) + {\mathrm{H}}_{{2}} {\mathrm{O}}\left( {\mathrm{g}} \right) = {\mathrm{CO}}\left( {\mathrm{g}} \right) + {\mathrm{H}}_{{2}} \left( {\mathrm{g}} \right) $$

The extent and location of these reactions strongly affect coke porosity development and strength loss.^69,70^ A recent review further emphasized that H_2_O generated under H₂-rich BF conditions can become an important gasifying agent and accelerate coke structural degradation, making H_2_O control critical for maintaining coke integrity.^71^ Shin and Jung^69^ observed that, compared with CO_2_, involving H_2_O (g) in the gasification process, the reaction was more likely to occur near the surface of the coke, which led to a more significant decrease in the strength of the coke. Therefore, controlling the generation of CO_2_ and H_2_O (g) in the high-temperature region is crucial for maintaining coke integrity and its supporting role in the furnace. Lan et al.^72^ found from thermodynamic calculations and experimental results that increasing H_2_ content in the reducing gas could promote the reduction of iron oxides to lower temperature regions and reduce the generation of CO_2_ in the high-temperature zone. When *φ*(CO)/*φ*(H_2_) decreases, the main carbon gasification temperature zone becomes narrow and moves downward in the BF, effectively weakening coke solution loss in the furnace bosh and cohesive regions.^72^ This trend is consistent with coupled coke gasification and sinter reduction experiments in CO-N_2_-(25%)H_2_ atmospheres, which showed that the enrichment of H_2_ changed the evolution of the carbon structure of coke and the formation process of pores. With less CO_2_ produced and a modified reaction pathway, H_2_-rich conditions can weaken coke structural degradation and help retain more post-reaction strength under moderate H_2_ levels.^73,74^ Thus, H_2_-rich operation indirectly stabilizes the coke structural role by relocating and weakening solution loss reactions rather than strengthening coke directly. The key finding here is that H_2_ can lower coke degradation not because coke becomes intrinsically stronger, but because the reaction environment becomes less destructive to coke in the most critical furnace zones.

#### Indirect Reduction with H_2_

Hydrogen reduction of iron oxides proceeds via:^75^

3 $$ {\mathrm{FeO}}\left( {\mathrm{s}} \right) + {\mathrm{H}}_{{2}} \left( {\mathrm{g}} \right) = {\mathrm{Fe}}\left( {\mathrm{s}} \right) + {\mathrm{H}}_{{2}} {\mathrm{O}}\left( {\mathrm{g}} \right) $$

Compared with CO, H_2_ has higher diffusivity and generally faster reaction kinetics, enhancing indirect reduction rates in the shaft region of the BF.^76–78^ Numerical simulations by Mauret et al.^15^ showed that intensive H_2_ injection, via coke oven gas, natural gas, or pure H_2_, can increase the fraction of indirect reduction by H_2_ at the expense of CO-based reduction, thereby lowering the carbon demand per mole of oxygen removed, significantly reducing the coke rate, and maintaining stable furnace operation within acceptable process limits. Okosun et al.^79^ carried out CFD analysis and reported that up to ~ 35 kg H_2_/tHM injection can reduce coke consumption by approximately 60 kg/tHM under controlled flame temperature conditions. Zhao et al.^80^ further found that shaft injection of reformed coke oven gas could enhance near-wall reduction degree, narrow the cohesive zone, and decrease coke rate through improved H_2_ utilization efficiency. Similarly, a multi-fluid BF modeling was performed by Yu and Shen,^81^ which indicated that H_2_ enrichment could promote earlier reduction in the upper shaft, facilitate the cohesive zone upward, and increase gas utilization efficiency.

However, H_2_-based reduction also presents several challenges. H_2_ reduction may suffer from diffusion limitations in the later stages of iron oxide reduction, which can hinder complete metallization and influence overall process efficiency.^64^ Therefore, although H_2_ can promote indirect reduction and lower coke consumption, these kinetic limitations must be considered when evaluating the H_2_-enhanced ironmaking process. Overall, the benefit of H_2_-rich operation lies in increasing indirect reduction efficiency and lowering carbon demand, but the extent of substitution is still constrained by kinetics, thermal balance, and modeling uncertainties related to gas utilization, temperature distribution, and operational limits.^82^

#### Behavior of Water–Gas Shift Reaction

A critical aspect of H_2_-rich operation is the water–gas shift reaction:^83^

4 $$ {\mathrm{CO}}\left( {\mathrm{g}} \right) + {\mathrm{H}}_{{2}} {\mathrm{O}}\left( {\mathrm{g}} \right) = {\mathrm{CO}}_{{2}} \left( {\mathrm{g}} \right) + {\mathrm{H}}_{{2}} \left( {\mathrm{g}} \right) $$

H_2_ enrichment increases H_2_O generation through H_2_-based indirect reduction (i.e., reaction 3), which in turn promotes the above water–gas shift reaction. As a result, the balance among CO, CO_2_, H_2_, and H_2_O is changed, directly influencing both CO and H_2_ utilization efficiencies.^15^ Kemppainen et al.^84^ carried out experimental studies on pellet beds and found that the water–gas shift reaction becomes both thermodynamically feasible and kinetically significant at 300–500°C, particularly in the presence of magnetite, which exhibits a catalytic effect. Nogami et al.^85^ also investigated the effect of this water–gas shift reaction by numerical simulation, further demonstrating that this reaction plays a decisive role in the formation of the gas composition field, and therefore should be considered in H_2_-intensive BF models. Thus, proper control of the water–gas shift reaction enables improved CO efficiency and enhances overall gas utilization. However, the increased H_2_O content under H_2_-rich conditions may also accelerate coke gasification through the C-H_2_O reaction, leading to coke solution loss, pore development, strength degradation, and possible deterioration of burden permeability. The extent of this effect depends on the local H_2_O partial pressure, temperature, residence time, coke reactivity, and gas-flow distribution. Although the direct effect of H_2_O on oxide furnace materials is usually less significant than slag/metal corrosion, the associated change in local oxygen potential should still be considered in H_2_-rich BF operation.

H_2_ injection is also constrained by its impact on the thermal state of the lower furnace.^86–88^ Recent modeling of the raceway adiabatic flame temperature indicates that H_2_-rich gas injection must be coupled with suitable oxygen enrichment, blast-temperature control, and injection-rate optimization to maintain an acceptable tuyere-raceway temperature.^89^ This is because H_2_-related reactions are strongly endothermic, and excessive H_2_ addition can lower the flame temperature and may destabilize the lower furnace.^79^ Recent numerical simulations further show that H_2_ and pulverized coal co-injection, as well as the interaction between H_2_ injection and oxygen enrichment, strongly affect PCI combustion, raceway gas composition, and temperature distribution.^90,91^ Moreover, the type and injection amount of H₂-rich gas, together with natural gas and pulverized coal co-injection strategies, determine the feasible operation window for reducing carbon emissions while maintaining raceway stability and lower-furnace heat balance.^92,93^ In addition, numerical simulations of biomass- and coke oven gas-derived H_2_-rich fuels co-combusted with pulverized coal further suggest that alternative fuel injection should be evaluated together with combustion efficiency, temperature distribution, and lower-furnace thermal stability.^94^ Similar thermodynamic limitations have also been reported for CH_4_ injection, where excessive endothermic reaction reduces the heat supply to the lower zones.^95^ In this case, top gas recycling oxygen BF systems are proposed which enable H_2_-rich operation by recycling CO-rich streams, thereby reducing overall carbon demand, fuel rate, and CO_2_ emissions while maintaining thermal balance.^96,97^ More recently, Zhang et al.^98^ investigated the effect of H_2_-rich gas composition on energy consumption and raceway state in an oxygen BF, further showing that the composition and injection strategy of H_2_-rich gas must be carefully controlled to balance carbon reduction, energy consumption, and raceway stability. Furthermore, Babich et al.^99^ clarified that the optimization of H_2_ injection distribution at the shaft and tuyere could effectively reduce the coke ratio, while maintaining the stability of raceway temperature and cohesive zone, thereby achieving a higher H_2_ utilization rate. Therefore, the practical value of H_2_-rich BF operation depends not only on reduction efficiency, but also on whether gas composition, heat balance, and lower-furnace stability can be simultaneously maintained.

#### Industrial Demonstrations and Practical Limits of H_2_-Rich BF Operation

In addition to laboratory studies and numerical simulations, several industrial or near-industrial demonstrations have provided useful evidence for the practical implementation of H_2_-rich BF operation. For example, H_2_ injection was tested in an operating BF at the Duisburg site of Thyssenkrupp Steel,^100,101^ where H_2_ was injected through tuyeres as a partial replacement for pulverized coal. These tests provided important operational information on lance position, flow and pressure conditions, and the interaction between H_2_ injection and high-temperature BF operation. In Japan, the COURSE50 project has further investigated H_2_ injection into BFs using coke oven gas, room-temperature H_2_, and heated H_2_ in experimental BF systems.^102^

These demonstrations confirm that H_2_-rich BF operation is technically feasible and can reduce the carbon demand associated with iron oxide reduction. However, they also show that H_2_ injection should be regarded as a partial decarbonization route rather than a complete replacement for coke. The achievable benefit depends strongly on H_2_ availability and cost, tuyere and shaft injection strategy, oxygen enrichment, raceway adiabatic flame temperature, gas utilization efficiency, and the control of H_2_O-induced coke gasification. Moreover, H_2_ injection mainly replaces part of the chemical function of coke and does not directly replace its load-bearing and permeability-maintaining role in the cohesive zone and lower furnace. Therefore, industrial H_2_ BF demonstrations are valuable as bridge technologies for lowering coke consumption, but deeper coke reduction still requires simultaneous control of furnace thermal balance, burden permeability, and coke structural degradation.^15,101^

### Load-Bearing Packed-Bed Materials

Instead of replacing coke with reactive carbonaceous materials or using an H_2_-rich atmosphere, another approach is to introduce load-bearing packed-bed materials into the high-temperature zone of the BF, which can reduce the reliance on coke for mechanical support and maintain the permeability and structural stability under BF conditions.^103^ Potential materials must satisfy some critical requirements: (1) high-temperature mechanical strength under loading, (2) chemical stability with CaO-SiO_2_-Al_2_O_3_-MgO-FeO slags and molten iron, and (3) a sufficiently stable pore structure to maintain gas permeability. However, most existing studies focus on refractory or ceramic material performance in static corrosion environments rather than in typical dynamic slag-metal systems relevant to real BF operation. Therefore, the materials reviewed in following subsections should be regarded mainly as exploratory candidates that may provide guidance for future investigation. This route is conceptually important because it directly targets the structural role of coke, but it is also the least mature among all available approaches. The present discussion should therefore not be interpreted as evidence that ceramic or hollow packed-bed materials are ready for industrial BF application. Instead, these materials are reviewed to define the performance requirements and failure modes that must be addressed before any structural coke substitute can become realistic.

#### Oxide-Based Ceramic Systems

Porous oxide ceramics with high melting temperatures have been considered as potential load-bearing materials. Among them, MgO-Al_2_O_3_ materials are attractive due to their high melting temperatures and spinel-forming capability. Recent machine-learning-assisted optimization of multi-element spinel refractory materials also suggests that composition design can be used to improve mechanical properties, providing useful guidance for future development of spinel-based load-bearing ceramic candidates.^104^ Salomão et al.^105^ investigated porous alumina–spinel ceramics for high temperature applications and found that MgAl_2_O_4_-containing porous ceramics could retain a stable porous structure even after high-temperature treatment above 1300°C, which suggests the possibility of maintaining structural integrity at BF-related temperatures. However, their interaction with BF slags still requires careful consideration. Wang et al.^106^ combined thermodynamic calculations with experimental validation and showed that Al_2_O_3_-rich refractory materials could react with CaO-containing slags to form calcium aluminates such as CaAl_12_O_19_ and CaAl_4_O_7_. Interface investigations by Park et al.^107^ further revealed the formation of reaction layers including CaAl_12_O_19_ and Mg(Mn)Al_2_O_4_ spinels in CaO-CaF_2_-SiO_2_-Al_2_O_3_-MgO-MnO slags. While these reaction layers may sometimes act as diffusion barriers under static conditions, their stability under dynamic BF-relevant environments remains uncertain because slag flow, metal dripping, and thermal cycling may disrupt or dissolve the calcium aluminate layer. Therefore, Al_2_O_3_-MgO systems should be regarded as semi-inert materials with conditional interfacial protection rather than fully stable load-bearing substitutes.

ZrO_2_-based materials are also frequently investigated due to their extremely high melting temperature and potential for forming protective calcium zirconate layers. In high-basicity slag systems, ZrO_2_ can react with CaO to form CaZrO_3_ and related high-melting compounds that limit further erosion and reaction of the slag.^106^ However, Kumar et al.^108^ reported that, under oxidizing and MnO-rich slag conditions in ZrO_2_-C refractories, the consumption of phase-stabilizing Y_2_O_3_ from ZrO_2_ granules may result in their fragmentation and subsequent diffusion in slag-rich regions, eventually leading to weakening of their structural strength. In comparison, MgO-ZrO_2_ composite systems showed improved resistance in some metallurgical slags due to the formation of high-melting interfacial phases of ZrSiO_4_ and MgO-ZrO_2_-based stable solid solutions, which can reduce slag wettability and impede the penetration path.^109,110^ This suggests that composite oxide designs may provide better semi-inert performance than simple oxide systems in some cases, although their long-term behaviors under dynamic BF conditions still require further evaluation. In short, current research in oxide-based ceramic systems demonstrate that high-temperature stability alone is not sufficient, and the decisive issue is whether the material can retain both structure and chemical compatibility under continuous slag attack and abrasion.

#### Hollow Spheres and Engineered Spherical Materials

From a structural perspective, hollow ceramic spheres are among the material forms closest to porous load-bearing materials, where the particle size, the pore structure, and the permeability can be controlled through synthetic parameters. Qi et al.^111^ investigated hollow sphere ceramics and showed that, as the sintering temperature increased from 1400°C to 1600°C, the samples shrunk increasingly and the porosity decreased from 59% to 42%, which led to an increase in the strength of the alumina foams from 6.9 MPa (at 1400°C) to 100.0 MPa (at 1550°C). They further found that the mechanical strength of these materials was highly controlled by the contact area between the hollow spheres, which could be enlarged by increasing the sintering temperature, reducing the hollow sphere size or introducing slurry infiltration.

Hollow alumina sphere castables have also been explored to construct lightweight aggregate systems with low thermal conductivity and reasonable mechanical strength. Xu et al.^112^ studied hollow alumina sphere castables containing in situ-formed Y_3_Al_5_O_12_, and found that interfacial strengthening at the sphere–matrix interface improved the strength of individual particles, increased cold crushing strength and high-temperature modulus of rupture, and further reduced thermal conductivity. These studies suggest that interfacial engineering and multiphase design can help maintain mechanical performance under higher temperatures. The key insight is that structural design at the particle and interface levels can substantially improve strength; however, evidence under real BF conditions with slag and hot metal remains lacking.

#### Industrial Concepts from Cupola Furnace Systems

In addition to laboratory studies, several patents have proposed the use of engineered spherical materials to replace the structural role of coke in cupola furnaces rather than BFs. Ceramic balls prepared from Al_2_O_3_-SiO_2_-C-binder systems have been suggested as packed-bed materials to support the burden while maintaining gas permeability at high temperature.^113^ Some designs also proposed to employ high density carbon balls in modified cupola furnaces, where the high-carbon balls replace traditional water-cooled grates and provide structural support for the burden.^114^ Simultaneously, they also performed a critical carburizing function by enriching the iron droplets with carbon to achieve the desired final chemistry. Although such concepts are mainly proposed for cupola-type systems rather than conventional BFs, they still provide an alternative approach for using engineered spherical materials to reduce the dependence on traditional coke.

#### Critical Remarks on Load-Bearing Packed-Bed Materials

Although oxide ceramics, hollow spheres, and engineered spherical materials provide useful concepts for reducing structural coke dependence, their transfer into real BF operation faces major physical, chemical, and economic challenges. In the lower part of the furnace, molten slag and metal droplets, together with severe abrasion and thermal cycling, are present simultaneously. Under such conditions, pores or interparticle voids may act as pathways for slag infiltration, leading to blockage, weight gain, or structural disassembly. In addition to these physical challenges, chemical stability remains a further concern. Al_2_O_3_-based materials can react with CaO-rich slag, while ZrO_2_ may form protective calcium zirconate layers but can still degrade under BF conditions. Moreover, the behavior of materials is very different under dynamic abrasion and slag infiltration in real FeO-rich furnace environments.^115^

Beyond the technical feasibility, cost and large-scale production also present major barriers. The porous aggregates such as ZrO_2_ or spinel are much more expensive than coke and would need to be supplied continuously at large scale in the BF. Issues related to slag separation, recycling, and coexistence with the existing BF conditions also remain unresolved. Therefore, these materials are better regarded as exploratory options and a long-term research direction for structural substitution, rather than immediately practical low-carbon solutions for industrial BF ironmaking.

## Burden, Furnace Structure, and Operational Optimization as Indirect Coke-Saving Routes

An industrially feasible approach to reduce the coke content is not to replace the coke structural role directly, but to lower the mechanical and permeability demands through burden and furnace structure optimization. In addition to burden optimization, conventional operational measures also play important roles in reducing coke ratio in industrial BF practice. Increasing the hot-blast temperature can provide additional sensible heat to the lower furnace and reduce the amount of coke required for heat supply.^116,117^ Oxygen-enriched blast increases combustion intensity in the tuyere-raceway region, promotes pulverized coal burnout, and allows a higher auxiliary fuel injection rate.^118^ High-pressure operation can improve gas–solid contact and gas utilization efficiency, helping to stabilize the furnace and lower the coke rate, although its practical benefit depends on furnace condition and operational constraints.^119^ However, these measures mainly reduce coke consumption through thermal and gas-flow optimization, rather than directly replacing the load-bearing and permeability-maintaining function of coke. Therefore, they should be considered as complementary industrial measures for coke saving, while deeper coke reduction still requires simultaneous consideration of furnace stability, cohesive-zone permeability, and coke structural function.

The softening-melting behavior of burden materials plays a key role in determining cohesive zone thickness and gas permeability. Recent studies under H_2_-rich or hydrogen-enriched BF conditions further show that primary slag formation, carburization of metallic iron, and Al_2_O_3_-related slag characteristics can significantly affect cohesive-zone behavior and burden permeability.^120,121^ Chiu et al.^122^ showed under simulated BF conditions that grain coarsening of Fe and FeO and liquid phase evolution could strongly influence dripping behavior and permeability. Mixed-burden experiments further showed that interaction between acid and basic components above 1150°C could modify liquid formation and interfacial reactions, thereby affecting cohesive zone structure.^123–125^ In addition, optimization of MgO and Al_2_O_3_ contents in sinter or high-alumina burdens can adjust softening–melting intervals, slag characteristics, and permeability of mixed ferrous burdens.^121,126^ In the upper shaft region, Barustan et al.^127^ analyzed factors affecting the burden surface and porosity distribution, and showed that H_2_-containing gases might enhance gas diffusion into particles and promote the hematite–magnetite transformation. Depending on burden type, this can induce internal cracking, alter degradation behavior, and change gas permeability. These effects also influence fines generation and therefore affect the level of structural support and permeability control required from coke.

From a structural perspective, burden distribution control is another critical concern. Simulation studies carried out by Wei et al.^128^ and Roeplal et al.^129^ showed that the material charging approach could strongly affect the radial distribution of burden materials, which relates to bed porosity and permeability. These changes redistribute the ascending gas flow and consequently influence the shape and position of the cohesive zone. Layered cohesive zone modeling by Dong et al.^130^ further confirmed that proper burden layering could form low-permeability ore layers and highly permeable coke layers, causing gas to flow preferentially through the coke layers and liquids to pass through these permeable paths in the lower furnace.

Overall, improving burden quality, optimizing softening–melting behavior, and adjusting charging practice can help maintain acceptable gas permeability and liquid flow even at lower coke rates. Under these conditions, more coke mainly acts as a permeability stabilizer and carburizer, rather than serving as the primary structural role, providing a practical route for low-carbon BF operation. This route is particularly important because it does not require a new substitute material for coke. Instead, it reduces the extent to which the furnace relies on coke by making the burden-furnace system itself easier to operate at lower coke rates. In that sense, burden and furnace optimization is likely to be one of the most practical near-term approaches for reducing coke dependence.

## CO_2_-to-CO Conversion as an Enabling Route for Low-Coke BF Operation

Recent studies suggest that part of CO required in a BF can be provided by self-generated CO_2_ rather than produced from coke, such as thermochemical CO_2_ splitting using BCNF-type perovskite materials and electrochemical CO_2_ conversion.^19–23^ BCNF-based systems can convert CO_2_ into CO through redox cycles at around 700–800°C, providing a potential approach to recycle CO_2_ within the ironmaking process.^22,23^ At the same time, CO_2_ electrolysis technologies have also been proposed to transform captured BF CO_2_ into CO-rich gas streams that can be re-injected into the furnace, which may significantly reduce fresh coke consumption while lowering overall CO_2_ emissions.^19,21^ In addition, CO_2_ utilization through co-injection with H_2_ into BF tuyeres has been evaluated as another possible route for carbon recycling and coke-rate reduction.^131^ These approaches suggest that CO used for iron oxide reduction does not necessarily have to come only from coke combustion but can also be regenerated from captured CO_2_.

However, even if part of the reducing gas is supplied from recycled CO or H_2_-rich gas streams, the BF still requires a solid carbonaceous material to maintain its internal structure. Therefore, most current low-carbon BF concepts focus on reducing rather than completely eliminating coke. This means that CO_2_-to-CO conversion should be understood as an enabling technology for lowering the chemical demand for coke, not as a standalone solution to the coke problem. Therefore, in actual industrial settings, the process toward lower coke consumption is usually achieved through a combination of several approaches. One option is the partial replacement of conventional coke with the above-mentioned carbonaceous materials, which can decrease the demand for fossil carbon while maintaining acceptable burden permeability. Another is the injection of H_2_-rich or CO-rich gases to increase the indirect reduction ratio and reduce the amount of carbon required for reduction reactions. In addition, optimization of burden composition and the cohesive zone behavior is also necessary to maintain stable gas flow and permeability when the coke rate is lowered.

**Fig. 2:**
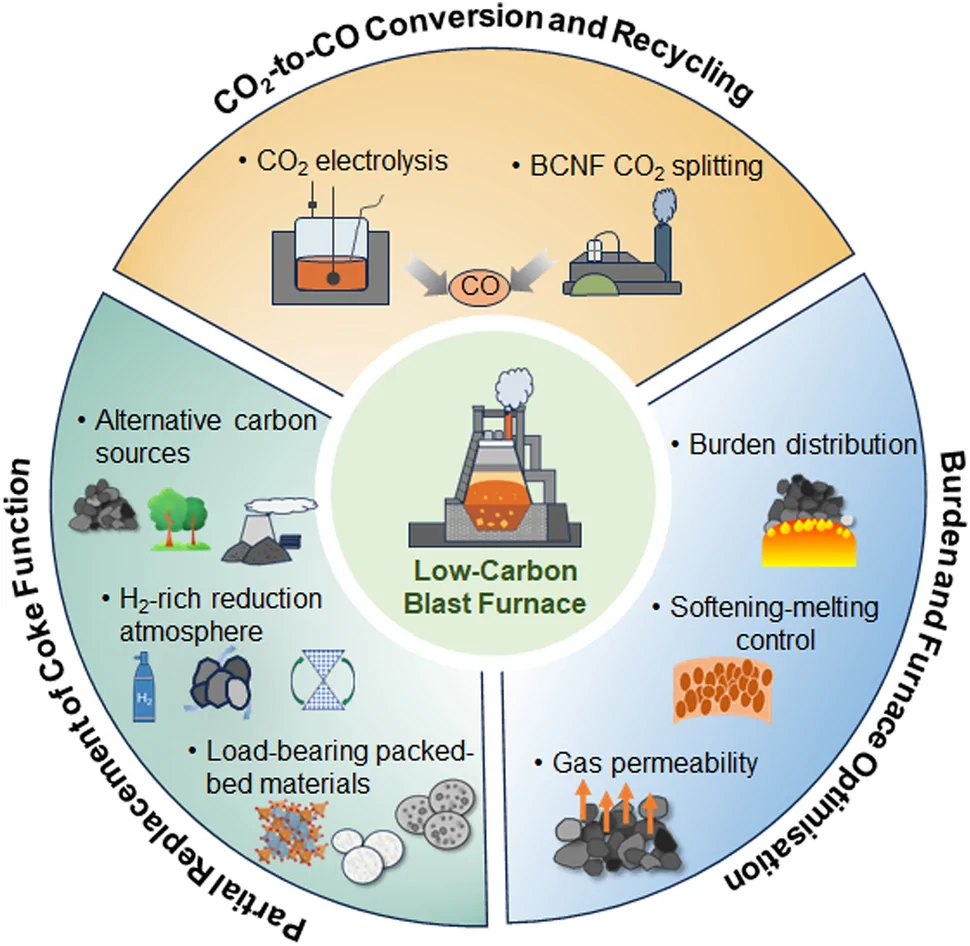
Overall strategies to reduce coke demand in low-carbon BF.

**Fig. 3:**
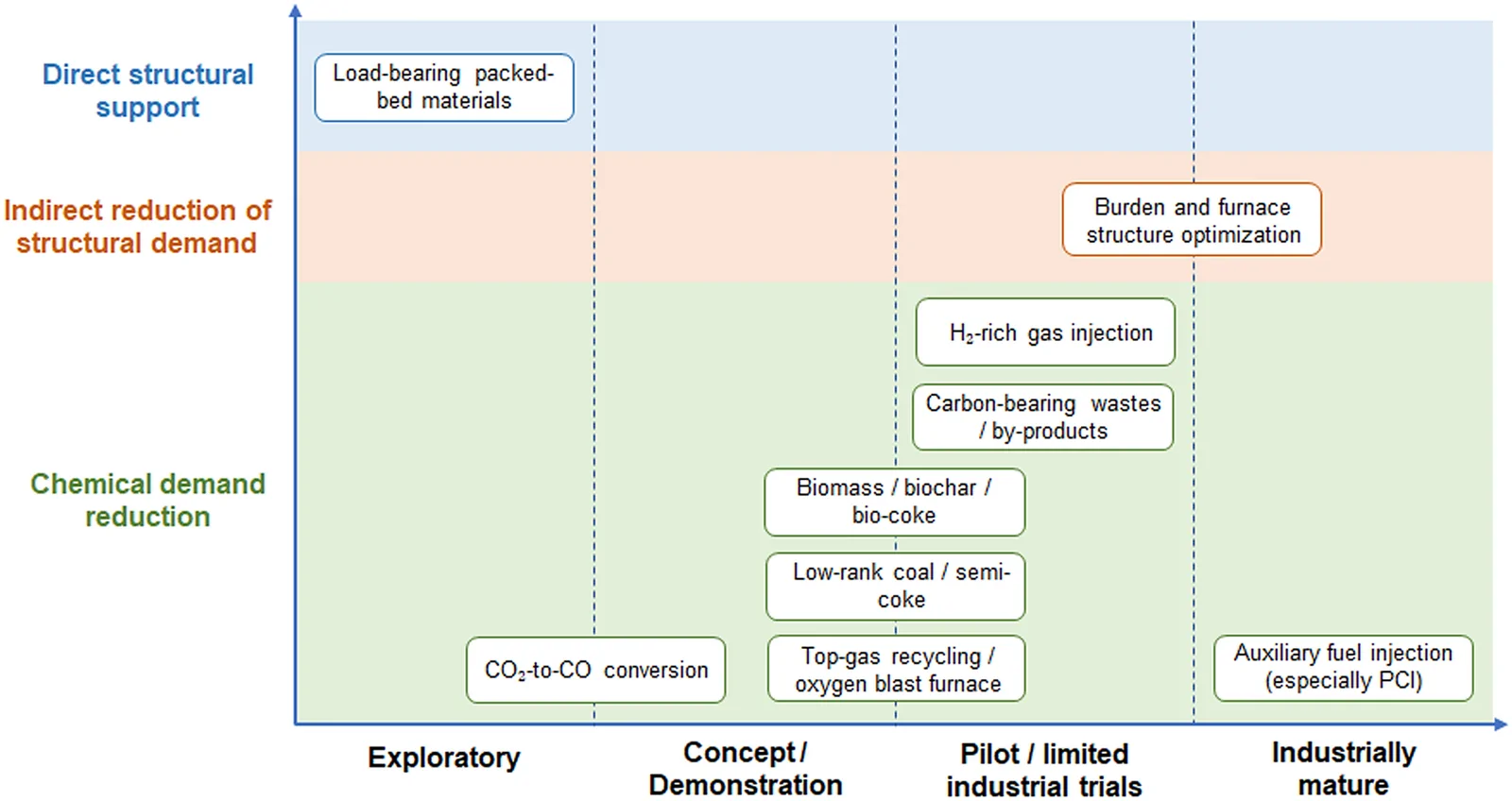
Relative maturity and principal route of different coke-reduction strategies.

In short, technologies such as BCNF-based CO_2_ splitting or CO_2_ electrolysis can be viewed as enabling approaches for lowering CO_2_ emissions and generating CO. By converting unavoidable CO_2_ emissions into effective CO, these technologies provide an additional source of reducing gas and help close the carbon loop within the BF system. When combined with H_2_ enrichment, recycled gas injection, and improved burden design, coke can gradually shift from being the main reductant to primarily serving as a structural support material and carburizer in the furnace. Thus, the overall strategy for reducing coke demand in a low-carbon BF can be considered as a comprehensive optimization of three aspects: potential materials for partial replacement of coke function, burden and furnace structure optimization, and CO_2_-to-CO conversion and recycling, as illustrated in Fig. 2. This suggests that meaningful coke reduction will likely require a combination of measures rather than relying on any single technology alone. Overall, these strategies do not reduce coke dependence in the same way. Some mainly lower the chemical demand for coke, while others help to reduce the structural dependence on coke. As shown in Fig. 3, the more mature options are generally those that reduce coke use indirectly. In contrast, direct replacement of coke’s structural role is still at an early stage.

## Overall Comparison of Coke-Reduction Strategies

### Functional Comparison of Coke-Reduction Strategies

Table I summarizes the main coke-reduction routes from the viewpoint of coke function, carbon-saving role, and key industrial limitations, while their relative maturity and functional positioning are further illustrated in Fig. 3. This comparison shows that different strategies should not be judged only by their theoretical carbon-saving potential. Their practical value depends strongly on which coke function they target and whether furnace stability can be maintained.

**Table I:** Critical comparison of representative coke-reduction routes according to coke function, carbon-saving role, and industrial limitations

Strategy	Main coke function targeted	Carbon-saving role	Key industrial limitation
Low-rank coal/semi-coke	Chemical demand reduction	Partial fossil carbon substitution	High reactivity; weak hot strength
Biomass/biochar/bio-coke	Chemical demand reduction	Lower net fossil CO_2_ input	Supply limitation, quality variation, weak structural role
Carbon-bearing wastes/by-products	Chemical demand reduction	Waste carbon recovery	Impurity accumulation, slag chemistry, permeability
Auxiliary fuel injection (especially PCI)	Chemical demand reduction	Reduces coke as fuel and reductant	Combustion efficiency, raceway stability, thermal balance
H_2_-rich gas injection	Chemical demand reduction	Lowers carbon-based reduction demand	H_2_ cost, thermal balance, H_2_O-induced coke degradation
Hot-blast temperature, oxygen enrichment, and high-pressure operation	Chemical demand reduction	Indirect coke saving	Limited by furnace stability and operating window
Top-gas recycling/oxygen blast furnace	Chemical demand reduction	High CO_2_-reduction potential	CO_2_ separation, O_2_ demand, energy penalty, system integration
Burden and furnace structure optimization	Indirect reduction of structural demand	Maintains permeability at lower coke rate	Ore quality, softening-melting control
CO_2_-to-CO conversion	Chemical demand reduction	Reuses captured CO_2_ as reducing gas	Conversion efficiency, energy demand, reactor integration
Load-bearing packed-bed materials	Direct structural support	Potential structural coke replacement	Slag attack, abrasion, cost, recyclability, large-scale supply

The comparison shows that current coke-reduction strategies should not be evaluated on the same basis. Alternative carbon sources, biomass-derived materials, and carbon-bearing wastes mainly reduce fossil carbon input, but their high reactivity, weaker hot strength, or impurity burden limits their ability to replace the structural function of coke. H_2_-rich reduction and recycled CO injection can reduce the chemical demand for carbon and improve gas utilization, but they still require a stable coke bed to maintain permeability, liquid drainage, and cohesive-zone stability. Industrial operational measures, such as hot-blast temperature, oxygen enrichment, high-pressure operation, and burden optimization, are more mature and can reduce coke rate indirectly, but their effects are limited by the thermal and permeability window of the BF. In contrast, load-bearing packed-bed materials directly target the most difficult structural role of coke, but this route remains exploratory because such materials must withstand simultaneous slag attack, metal dripping, abrasion, thermal cycling, and large-scale economic constraints. Therefore, meaningful coke reduction in BF ironmaking is more likely to be achieved by combining mature chemical-substitution and operational-optimization routes, while treating structural coke replacement as a longer-term research challenge.

### Techno-Economic and Implementation Considerations

Besides technical feasibility, the practical deployment of coke-reduction strategies must also be assessed from a techno-economic perspective. Mature routes such as PCI optimization, hot-blast temperature control, oxygen enrichment, high-pressure operation, and burden distribution optimization are more attractive for near-term application because they can be integrated into existing BF systems with limited reconstruction. In contrast, H_2_-rich operation, top-gas recycling/oxygen BF systems, and CO_2_-to-CO conversion require additional infrastructure for hydrogen supply, oxygen production, CO_2_ separation, compression, reheating, and re-injection, which may introduce extra energy supply and costs. Therefore, near-term coke reduction is more likely to rely on mature operational optimization and partial chemical substitution, while deeper coke reduction will require coordinated development of low-carbon energy supply, gas recycling, carbon management, and new materials capable of maintaining furnace permeability.

## Conclusion and Perspective

This review clarifies coke reduction not simply as a fuel-substitution problem, but as a functional substitution problem. From this perspective, most existing low-carbon strategies mainly reduce the chemical demand for coke, whereas the physical requirement for coke as a load-bearing and permeability-maintaining material remains the key bottleneck for deeper coke reduction. This review has examined several strategies currently being explored for low-carbon BF ironmaking, including alternative carbonaceous materials, H_2_-rich reducing gases, recycled CO derived from CO_2_ conversion, load-bearing packed-bed materials, and burden structure optimization. Alternative carbon sources, H_2_-rich gases, and recycled CO can contribute to lowering fossil carbon input and improving gas utilization. However, they do not fully replace the structural role of coke in the lower furnace and cohesive zone. Candidate load-bearing materials have been proposed to address this issue, but their practical application still faces major challenges in chemical compatibility, slag reactivity, mechanical degradation, cost, and large-scale supply.

### Main Conclusions

From this review, several main conclusions can be drawn:

- Alternative carbonaceous materials, including low-rank coal and semi-coke, and carbon-bearing wastes, are primarily effective as partial substitutes for the chemical function of coke, but generally cannot provide equivalent high-temperature structural performance;
- H_2_-rich operation can reduce coke demand indirectly by promoting H_2_-based indirect reduction and weakening coke solution loss, although its application remains constrained by gas-reaction complexity, reduction kinetics, and lower-furnace thermal balance;
- Load-bearing packed-bed materials are attractive because they aim to replace the structural role of coke directly, but they are still at an exploratory stage owing to unresolved issues of slag attack, abrasion resistance, cost, and large-scale application;
- Burden and furnace structure optimization offers a particularly practical route for lowering coke dependence because it reduces the structural burden placed on coke without requiring a fully new substitute material;
- CO_2_-to-CO conversion technologies should be regarded as supportive approaches for carbon recycling and gas re-utilization, rather than complete solutions for coke replacement.

### Perspectives

From an industrial perspective, reducing coke consumption is more likely to depend on the combination of several approaches than on any single technology. In this context, partial replacement of coke functions, burden and furnace optimization, and CO_2_-to-CO conversion can work together in a complementary way. Under such conditions, the role of coke may gradually shift from being the main reductant and structural support to mainly maintaining permeability, burden support, and process stability. However, the practical value of any low-coke strategy ultimately depends on whether carbon reduction can be achieved without affecting furnace productivity, thermal efficiency, and operational stability.
